# Application of Cytochrome C-Related Genes in Prognosis and Treatment Prediction of Lung Adenocarcinoma

**DOI:** 10.1155/2022/8809956

**Published:** 2022-10-03

**Authors:** Min Tang, Guoqing Li, Liang Chen, Jing Tu

**Affiliations:** ^1^Department of Oncology, Chongqing General Hospital, Chongqing 401147, China; ^2^Department of Pulmonary and Critical Care Medicine, Chongqing General Hospital, Chongqing 401147, China; ^3^Intensive Care Unit, Chongqing General Hospital, Chongqing 401147, China

## Abstract

Lung adenocarcinoma (LUAD) is the most common subtype of nonsmall cell lung cancer. Cytochrome c (Cyt c), which is produced from mitochondria, interacts with a protein called Apaf-1 to form the heptameric apoptosome. This heptameric apoptosome then activates the caspase cascade, which ultimately results in the execution of apoptosis. The purpose of our research was to discover a new prognostic model that is based on cytochrome c-related genes (CCRGs) for LUAD patients. Through LASSO regression analysis conducted on the LUAD datasets included in the TCGA datasets, a CCRGs signature was created. The diagnostic accuracy of the multigene signature was verified by an independent source using the GSE31210 and GSE72094 datasets. The GO and KEGG enrichment analysis were performed. In this study, there were 159 differentially expressed CCRGs in the TCGA dataset, while there were 68 differentially expressed CCRGs in the GSE31210 dataset. Additionally, there were 57 genes that overlapped across the two datasets. Using LASSO and Cox regression analysis, a signature consisting of 12 differentially expressed CCRGs was developed from the total of 57 such genes. On the basis of their risk ratings, patients were categorized into high-risk and low-risk categories, with low-risk patients having lower risk scores and a greater likelihood of surviving the disease. Univariate and multivariate analyses both concluded that this signature is an independent risk factor for LUAD. ROC curves demonstrated that this risk signature is capable of accurately predicting the 1-year, 2-year, 3-year, and 5-year survival rates of patients who have LUAD. The infiltration of antigen-presenting cells was higher in the low-risk group, such as aDCs, DCs, pDCs, and iDCs. The expression of multiple immune checkpoints was significantly higher in the low-risk group, such as BTLA, CD28, and CD86. Finally, we showed that the signature can be used to predict the drug sensitivity of already available or under investigational drugs. Overall, patient classification and individualized therapy options may benefit from this study's development of a powerful gene signature with high value for prognostic prediction in LUAD.

## 1. Introduction

One of the most frequent forms of cancer is the lung cancer which has reached epidemic proportions in recent years [1]. It poses a significant threat to human health and quality of life, ranking third most frequent behind breast and prostate cancers [2, 3]. Researchers studying cancer all over the world have been concentrating their efforts on the lung cancer. Nonsmall cell lung cancer (NSCLC) is the most common histological subtype of lung cancer and accounts for roughly 85 percent of newly diagnosed cases [4, 5]. Lung adenocarcinoma, also known as LUAD, is a subtype of NSCLC that has a high morbidity and mortality rate. In recent years, there have been considerable advancements in the treatment choices for persons who have been diagnosed with LUAD [6, 7]. These options include chemotherapy, radiation, and immunotherapy. Despite this, there is still a percentage of patients who have distant metastases and are unable to be treated effectively at an early stage due to a lack of sensitive biomarkers, resulting in poor 5-year survival rates [8, 9]. Hence, it is of the utmost need to find more efficient biomarkers for early diagnosis, therapy, and evaluation of prognosis.

A large protein complex known as the “apoptosome” is formed when cytochrome c (Cyt c) is released from mitochondria and interacts with an adaptor protein called apoptotic protease activating factor-1 (Apaf-1) [10, 11]. This complex then recruits and activates caspase-9, which begins the caspase cascade and the process of apoptosis. Thus, in the process of apoptosome-mediated caspase activation, the interaction between Cyt c and Apaf-1 is a crucial upstream event [12]. The “Warburg effect” describes the tendency of most cancer cells to rely more heavily on aerobic glycolysis rather than OxPhos as their primary source of energy [13, 14]. Cancer cells must go through the process of metabolic reprogramming in order to boost anabolic biomass production, which is necessary for cell growth. Cancer is characterized by a number of key features, including dysregulation of cellular energetics and resistance to cell death. Cyt c is a protein that sits at the crossroads of several different pathways that can lead to both of these different carcinogenesis mechanisms [15, 16]. The significance of Cyt c in tumor angiogenesis, cell proliferation, as well as cellular differentiation and apoptosis has been shown by previous research. Nevertheless, the possible regulatory mechanism it possesses is not completely understood. In the past 20 years, there has been a recent uptick in research on the microenvironment of cancers, namely how immune cells play a pivotal part in the development of cancer. Cyt c has been shown in previous research to be able to influence the status of a tumor's immune microenvironment in a number of distinct ways, including encouraging the recruitment of innate immune cells and inhibiting the differentiation and functions of adaptive immune cells [17, 18]. Thus, it is necessary to conduct additional research on the link between Cyt c and immunity in LUAD in order to come up with innovative approaches to the treatment.

In our work, our group is aimed at thoroughly examining the roles of cytochrome c-related genes in LUAD and at developing a novel model based on cytochrome c-related gene signature. We anticipate that the findings of our research will provide a more in-depth understanding of the role that cytochrome c-related genes play in LUAD.

## 2. Methods and Materials

### 2.1. Data Acquisition

The transcriptome data of 535 LUAD and 59 para-tumor samples were retrieved from TCGA datasets. The clinical data and mutation data were also downloaded for survival analysis and tumor mutation burden (TMB) analysis, respectively. The GSE31210 dataset consisted of 226 tumor samples and 20 nontumor samples was obtained from GEO datasets. The GSE72094 dataset was downloaded as validation dataset, including 442 LUAD patients. The cytochrome c-related genes (CCRGs) were downloaded from GeneCards database (https://www.genecards.org), and 781 genes were screened for subsequent analysis according to the relevance score greater than 10 (Table S1).

### 2.2. Functional Enrichment Analysis of Differentially Expressed CCRGs

The differentially expressed CCRGs of TCGA dataset and GSE31210 were examined by the use of FDR < 0.05 and |logFC|>1 using ‘limma' package [19]. Subsequently, the ‘Venn' package used to find out their intersection genes. The GO analysis was conducted in Metascape (https://metascape.org). The KEGG analysis was conducted using ‘clusterProfiler' package [20].

## 3. Construction of a CCRGs Signature

A univariate Cox regression analysis was used to identify prognosis-related CCRGs utilizing the TCGA dataset as a training cohort. To create the prognostic CCRGs signature, we used LASSO Cox regression analysis, an approach that can prevent over fitting. The signature was as risk score = *e*^sum(eachgene'snormalizedexpression × eachgene'scorrespondingcoefficient)^. Patients were sorted into high-risk group and low-risk group as the median score of CRGs signature was the threshold.

### 3.1. Verification of CCRGs Signature Performance

Using the GSE31210 and GSE72094 as testing cohorts, the contents of verifying the performance including survival analysis, ROC curve, PCA, and t-SNE analysis were performed. Univariate and multivariate assays were carried out to explore the independent performance.

### 3.2. Immune Microenvironment and the CCRGs Signature

By the use of the ESTIMATE algorithm, the enrichment degree of immune cells and stromal cells in each sample was calculated [21]. The single sample gene set enrichment analysis (ssGSEA) was conducted to estimate the enrichment scores of sixteen immune cells to explore the relationship between the signature and immune cells using ‘GSEABase' package [22].

### 3.3. Immunotherapy and the CCRGs Signature

The relationship between the signature and immune checkpoints was also explored to explore whether immune checkpoint blockade (ICB) was different in different groups of patients. Tumor Immune Dysfunction and Exclusion (TIDE) algorithm could predict the effect of different groups of patients on anti-PD-1 and anti-CTLA-4 immunotherapy. The TIDE score was negatively correlated with the response effect of ICB treatment.

### 3.4. Tumor Mutation Burden and the CCRGs Signature

Higher TMB was associated with better ICB outcomes. We compared the difference in TMB between the two risk groups to predict the effect of ICB therapy. The mutation of CCRGs was explored on the cBioPortal database (http://www.cbioportal.org).

### 3.5. Chemotherapy and the CCRGs Signature

The CellMiner (https://discover.nci.nih.gov/cellminer) is a public database resource that provides drug sensitivity information files. We downloaded drug sensitivity information files from it and selected gene targeting drugs, which were approved by the Food and Drug Administration (FDA). The *P* value was shorted from small to large, and the first 16 analysis results were visualized.

### 3.6. Statistical Analysis

All statistical analyses were performed by R software (Version 4.2.1). A *P* value less than 0.05 was considered statistically different. Student's *t*-test and one-way ANOVA were, respectively, employed to evaluate two or multiple groups, for statistical significance. Differences in LUAD patient survival were assessed using the Kaplan-Meier method. A Cox regression analysis was adopted to assess the prognostic factors. Differences were considered statistically significant when *P* < 0.05.

## 4. Result

### 4.1. Differentially Expressed CCRGs and Functional Enrichment

**Figure 1:**
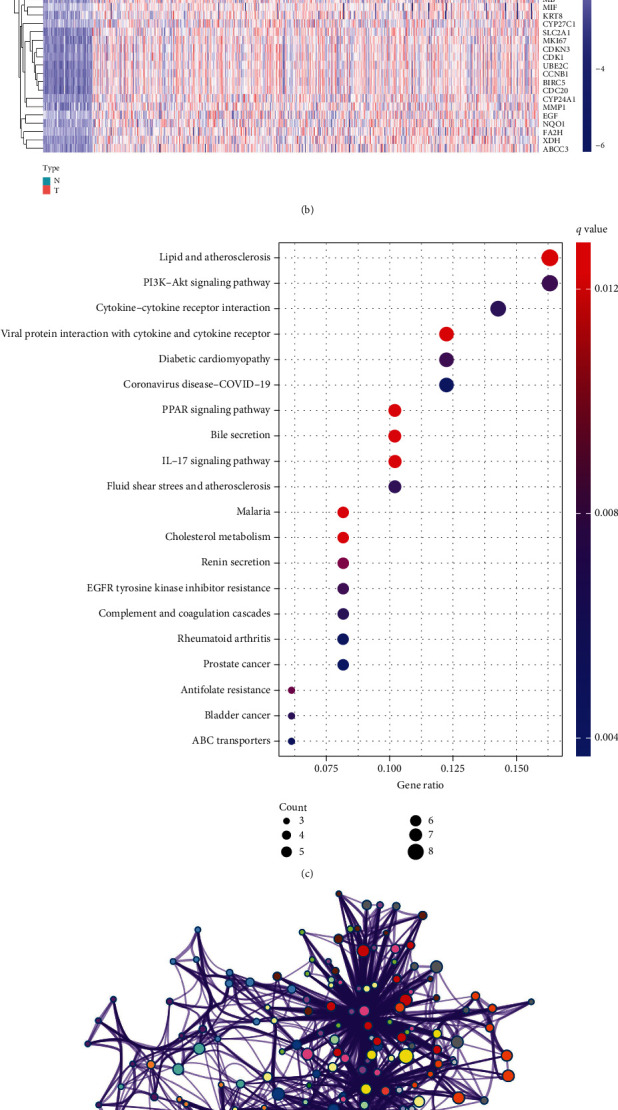
The expression and function enrichment of differentially expressed 57 CCRGs. (a) Venn Diagram. (b) Heatmap. (c) KEGG analysis. (d) GO analysis.

There were 159 differentially expressed CCRGs in TCGA dataset and 68 differentially expressed CCRGs in GSE31210, and there were 57 intersect genes (Figure 1(a)). The heatmap was used to show the differential expression of these 57 genes (Figure 1(b)). The KEGG analysis showed that 57 genes were enriched in PI3K-Akt signaling pathway, PPAR signaling pathway, EGFR tyrosine kinase inhibitor resistance, and so on (Figure 1(c)). GO assays revealed that 57 genes were participated in lung fibrosis, regulation of lipid localization, cellular response to lipid, and so on (Figure 1(d)). Our data may indicate that 57 genes were involved in the development of lung diseases and were related to EGFR-TKI treatment of lung cancer.

### 4.2. Construction of the CCRGs Signature

**Figure 2:**
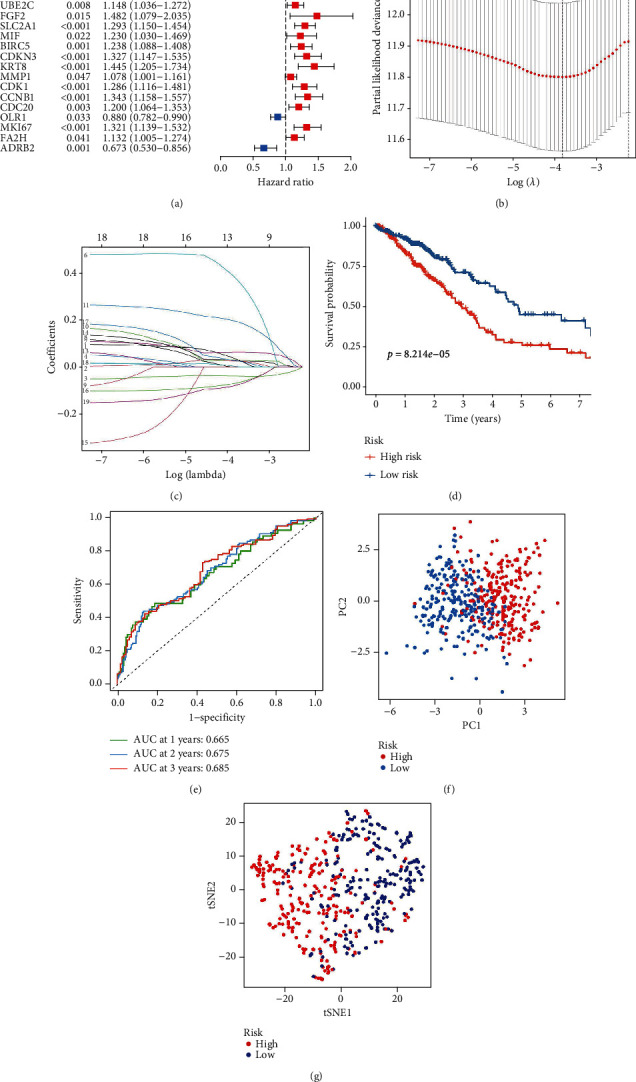
The construction of the CCRGs signature using TCGA training cohort. (a) Screening prognosis-related genes. (b) The process of selecting the appropriate penalty parameters. (c) The process of selecting 12-CCRGs to construct the signature. (d) The survival analysis. (e) The ROC curve. (f) PCA. (g) The t-SNE analysis.

Of the 57 differentially expressed CCRGs, 20 genes were associated with prognosis (Figure 2(a)). Finally, a 12-CCRGs signature was constructed using LASSO Cox regression analysis (Figures 2(b) and 2(c)). The risk score = (0.034772098∗CYP27C1) + (0.025435421∗CYP24A1) + (−0.038721339∗CYP4B1) + (0.378091608∗FGF2) + (0.063388606∗SLC2A1) + (0.030467492)∗CDKN3 + (0.193865279)∗KRT8 + (0.027231488∗CCNB1) + (−0.069261881∗OLR1) + (0.026932373∗MKI67) + (0.002432304)∗FA2H + (−0.078693326∗ADRB2). The survival analysis of TCGA dataset showed that the survival probability of high-risk group was distinctly lower than the low-risk group (Figure 2(d)). The AUC at 1, 2, and 3 years were 0.665, 0.675, and 0.685 (Figure 2(e)). PCA and t-SNE analyses demonstrated that high-risk group patients were clearly distinguished from low-risk group (Figures 2(f) and 2(g)).

### 4.3. Validation of the CCRGs Signature

**Figure 3:**
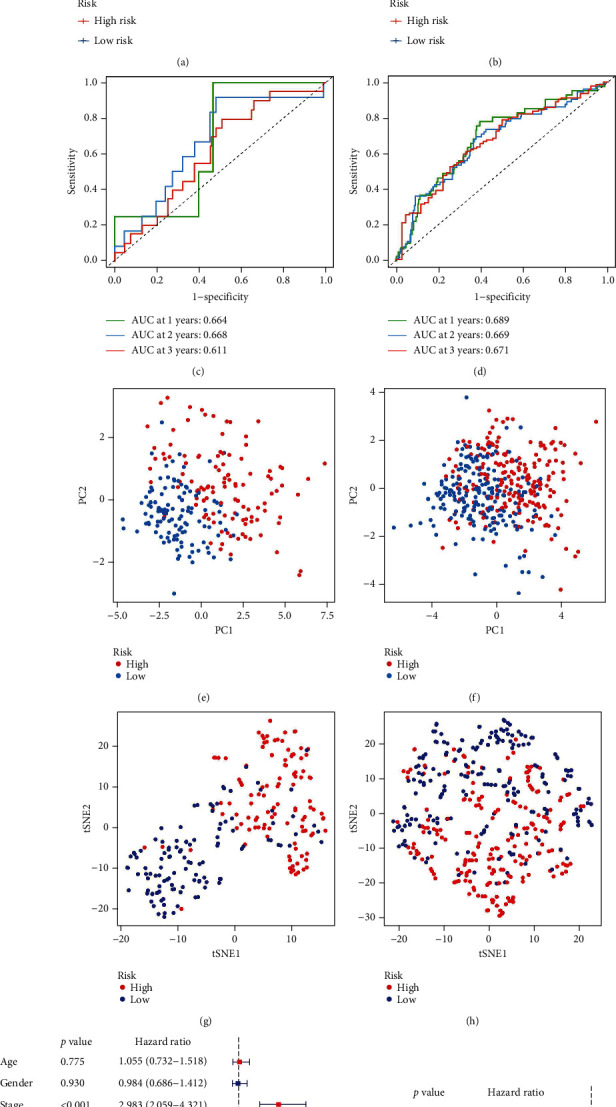
Validation of the CCRGs signature using two GEO datasets. (a) The survival analysis of GSE31210. (b) The survival analysis GSE72094. (c) The ROC curve of GSE31210. (d) The ROC curve of GSE72094. (e) PCA of GSE31210. (f) PCA of GSE72094. (g) The t-SNE analysis of GSE31210. (h) The t-SNE analysis of GSE72094. (i) Independence analysis using univariate Cox regression analysis. (j) Independence analysis using multivariate Cox regression analysis.

The GSE31210 and GSE72094 were used to validate the performance of CCRGs signature. The survival probability of high-risk groups in two testing datasets was lower than the low-risk groups (Figures 3(a) and 3(b)). The AUC of GSE31210 at 1, 2, and 3 years were 0.664, 0.668, and 0.611 (Figure 3(c)). The AUC of GSE72094 at 1, 2, and 3 years were 0.689, 0.669, and 0.671 (Figure 3(d)). The result of PCA and t-SNE analyses also showed that patients in two groups were clearly distinguished (Figures 3(e)–3(h)). What is more, the Cox assays were both showed that the CCRGs signature was an independent predictor (Figures 3(i) and 3(j)). The result all demonstrated that the 12-CCRGs signature had stable performance.

### 4.4. Immune Microenvironment and the CCRGs Signature

**Figure 4:**
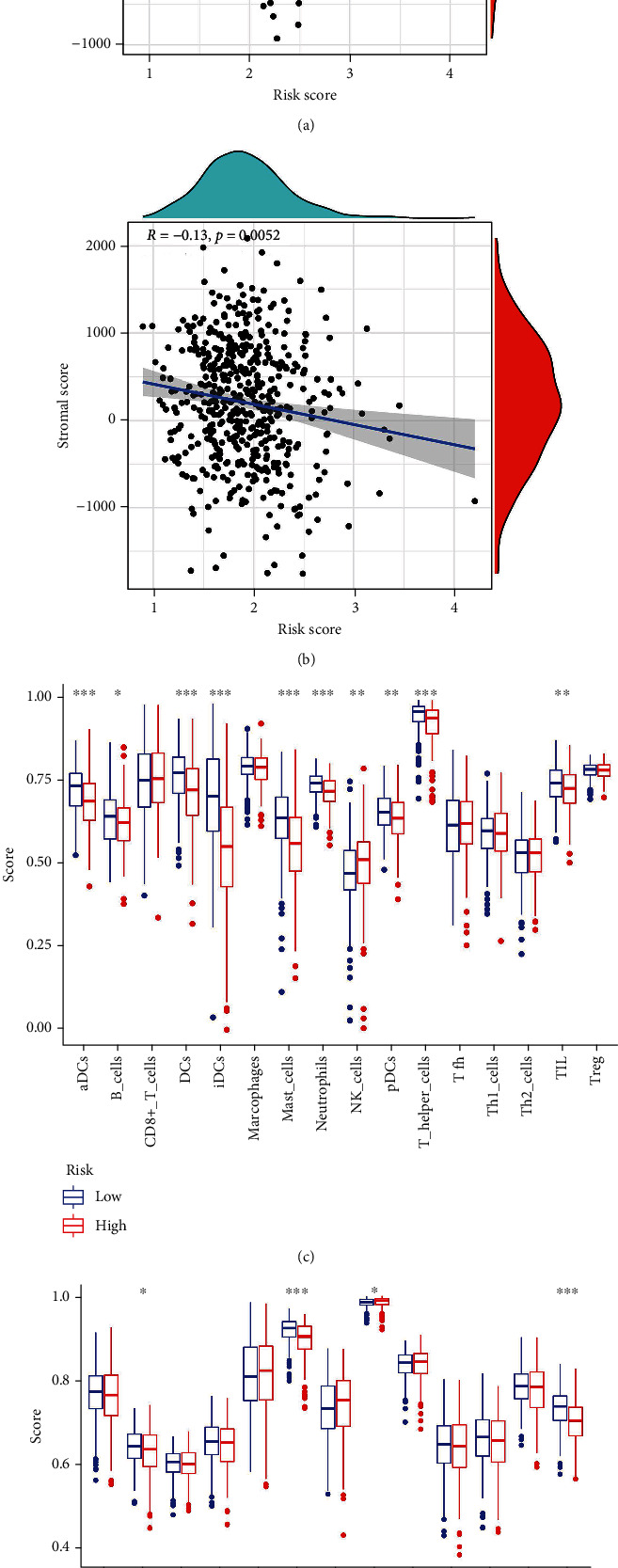
Immune related analysis of the CCRGs signature. (a) The infiltration of immune cells using ESTIMATE algorithm. (b) The infiltration of stromal cells using ESTIMATE algorithm. (c) The infiltration of immune cells using ssGSEA. (d) The analysis of immune related functions.

The infiltration degree of immune cells and stromal cells were negatively related to risk-score (Figures 4(a) and 4(b)), suggesting a distinct association between risk-score and immune microenvironment. The infiltration of antigen-presenting cells was higher in the low-risk group, such as aDCs, DCs, pDCs, and iDCs (Figure 4(c)). The infiltration of T helper cells and TIL was also higher in the low-risk group. Correspondingly, the antigen presentation process and HLA expression in the low-risk group were more active (Figure 4(d)).

### 4.5. Immunotherapy and the CCRGs Signature

**Figure 5:**
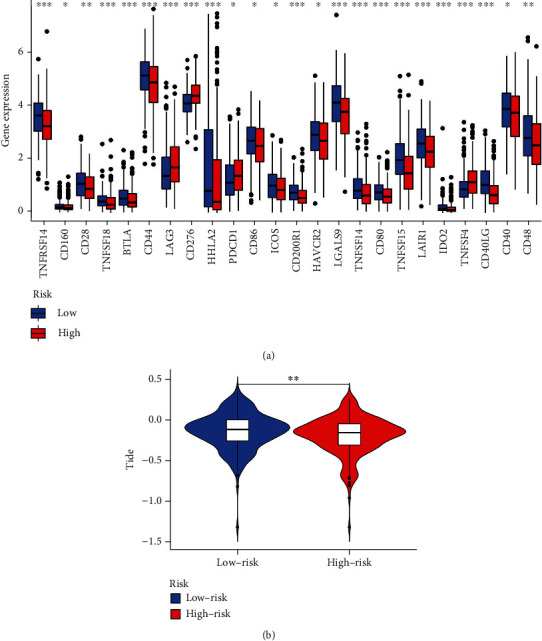
The application of the CCRGs signature in predicting the effect of immunotherapy. (a) The expression of immune checkpoint in predicting the effect of immunotherapy. (b) TIDE algorithm in predicting the effect of immunotherapy.

The expression of multiple immune checkpoints was significantly higher in the low-risk group, such as BTLA, CD28, and CD86. While the expression of PDCD1 (also known as PD-1) was higher in the high-risk group, it may indicate that the high-risk group had a better response to anti-PD-1 immunotherapy (Figure 5(a)). Further, the TIDE score of high-risk group was lower than the low-risk group, also indicating the high-risk group had a better response to anti-PD-1 or anti-CTLA4 immunotherapy (Figure 5(b)).

### 4.6. Tumor Mutation Burden and the CCRGs Signature

**Figure 6:**
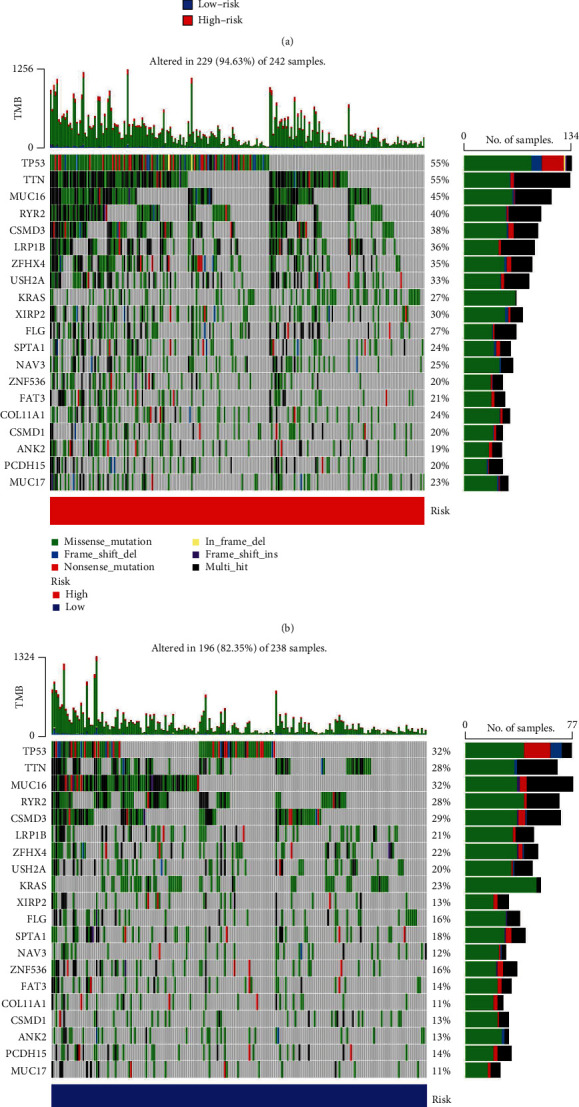
TMB and the CCRGs signature. (a) Comparison of TMB in two risk groups. (b) TMB of high-risk group. (c) TMB of low-risk group.

**Figure 7:**
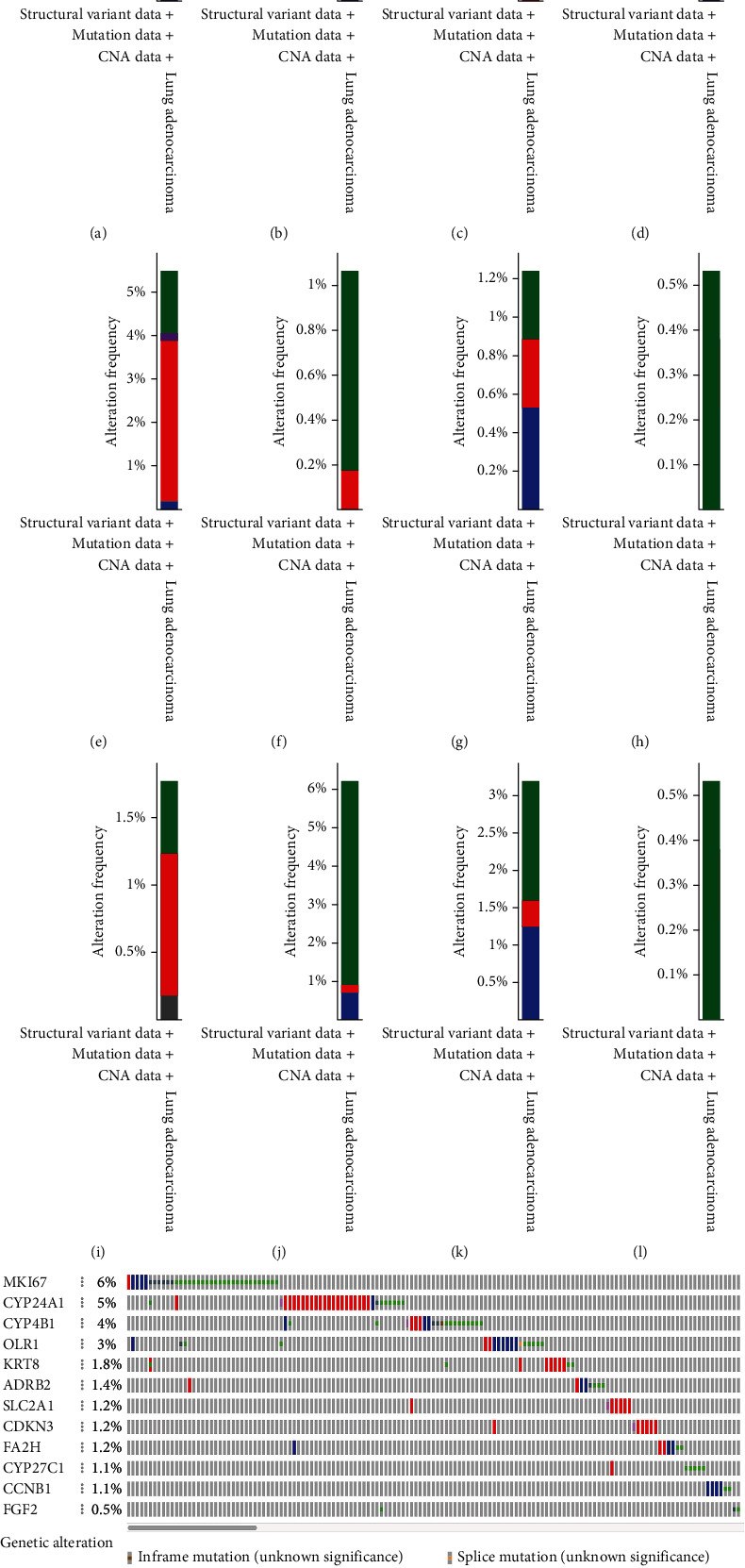
The mutation of 12 CCRGs. (a) ADRB2. (b) CCNB1. (c) CDKN3. (d) CYP4B1. (e) CYP24A1. (f) CYP27C1. (g) FA2H. (h) FGF2. (i) KRT8. (j) MKI67. (k) OLR1. (l) SLC2A1. (m) Summary of mutations in 12 CCRGs.

The TMB of high-risk group was 94.63% and significantly higher than the low-risk group, indicating that the high-risk group may have better ICB outcomes than the low-risk group (Figures 6(a)–6(c)). There were mutations in all 12 CCRGs (Figures 7(a)–7(m)) except FGF2, the common mutation type was amplification. The mutation rate of MKI67 was 6%, ranking the first at all 12 CCRGs, and the mutation rate of FGF2 was only 0.5%. Mutations in these genes may affect the effect of treatment.

### 4.7. Chemotherapy and the CCRGs Signature

**Figure 8:**
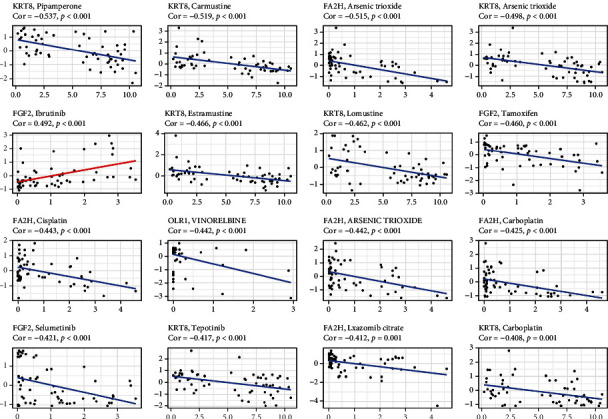
The application of the CCRGs signature in predicting the effect of chemotherapy.

Many of 12 signature CCRGs were sensitive to EGFR-TKI, such as CYP24A1, FA2H, FGF2, KRT8, and MKI67 were sensitive to Afatinib, ADRB2, CYP24A1, FA2H, FGF2, and MKI67 were sensitive to Dacomitinib, and SLC2A1, ADRB2, FGF2, and KRT8 were sensitive to Dasatinib. However, CYP27C1, CYP4B1, CDKN3, CCNB1, and OLR1 were resistant to many drugs. CDKN3 and CCNB1 were both resistant to Denileukin Diftitox (Ontak). CYP4B1 was resistant to Encorafenib, Pazopanib, Carmustine, and so on. CYP27C1 was sensitive to Ibrutinib, while it was resistant to Cobimetinib, Trametinib, Oxaliplatin, and so on. It was shown that there were no OLR1 sensitive drugs, but many resistant drugs, such as Sulfatinib, Paclitaxel, Vinblastine, and Vincristine (Figure 8).

## 5. Discussion

When it comes to health issues, lung cancer is ranked in second place, and it is the leading cause of death due to cancer in the entire world [23]. Nonsmokers are thought to have the highest prevalence of LUAD than smokers. Because of the proliferation of antismoking campaigns, the incidence of LUAD is quickly climbing to alarming levels [24, 25]. Even though there has been significant progress made in the treatment of cancer, the overall survival rate of LUAD patients continues to be unsatisfactory because there are no good early prognostic indications [26, 27]. More and more pieces of evidence have emerged in recent years linking CCRGs to the initiation and progression of various cancers. The results suggest a role for CCRGs in tumor development and progression. Therefore, to improve the outcomes of LUAD patients, it is absolutely necessary to locate reliable CCRGs markers. Because of this work, a predictive risk signature that is based on CCRGs has been successfully established for predicting the overall survival of patients with LUAD.

We firstly analyzed TCGA and GSE31210 to screen the differentially expressed CCRGs and identified 57 intersect genes. The KEGG analysis showed that 57 genes were enriched in PI3K-Akt signaling pathway, PPAR signaling pathway, EGFR tyrosine kinase inhibitor resistance, and so on, suggesting that the 57 differentially expressed CCRGs play an important role in tumor progression. After doing a LASSO Cox analysis, we isolated 16 CCRGs from these DEGs in order to construct a predictive signature consisting of 12 CCRGs. These genes had a role in the development of malignancies and had an impact on the prognosis of patients by recognizing and presenting antigens in the immune system. The function of the 12 CCRGs has been reported in several tumors, including LUAD. For instance, it was found that the degree of CCNB1 expression was clinically linked with a number of clinicopathological characteristics, such as gender, smoking status, tumor stage, and tumor stage. According to the findings of a survival analysis, a greater level of CCNB1 was associated with a more dismal outcome in terms of both overall survival and disease-free survival. In terms of its functionality, the degradation of CCNB1 by APC11 via UBA52 ubiquitylation was essential for the development of the cell cycle and the proliferation of NSCLC cell lines [28]. Mo et al. reported that the inhibition of CYP27C1 led to an increase in cell proliferation, migration, and invasion through the control of the signaling cascade involving IGF-1R, Akt, and p53 [29]. Xie et al. found that KRT8 is overexpressed in LUAD tissues, and its expression may be able to independently predict poor OS and RFS for LUAD patients, but not for LUSC patients. However, KRT8 is not overexpressed in LUSC tissues [30]. Xu et al. reported that in NSCLC cells, treatment with VEGFR2-TKIs led to an increase in the expression of ADRB2. By blocking the ADRB2 signaling pathway in NSCLC cells in vitro and in vivo, propranolol, a common ADRB2 antagonist, dramatically increased the therapeutic efficacy of VEGFR2-TKIs. This was demonstrated both in vitro and in vivo. Mechanically, NSCLC patients developed resistance to VEGFR2-TKIs as a result of the treatment-induced overexpression of ADRB2 and the strengthening of the interaction between ADRB2 and VEGFR2. Additionally, cells became more sensitive to VEGFR2-TKIs after the suppression of the ADRB2, CREB, and PSAT1 signaling pathway [31]. Overall, our findings suggested the 12 CCRGs served as tumor promotor or tumor suppressors.

All patients diagnosed with LUAD were split into two groups according to our model (high group and low group). According to the results of the survival tests, the new prognostic signature was able to assist medical professionals in classifying patients diagnosed with LUAD into two categories that have considerably different OS. The prognostic signature's ROC showed reasonable predictive accuracy in OS prediction for patients with LUAD, and it displayed good discrimination capacity of OS in subgroup analysis. The risk score of the prognostic signature was found to be capable of functioning as an independent prognostic factor after being subjected to multivariate Cox regression analysis. In addition, our findings were supported by further evidence found in the datasets GSE31210 and GSE72094.

Immunotherapy is a relatively new approach to cancer treatment that is receiving a growing amount of attention across a variety of cancer types, including LUAD [32]. However, the identification of patients who are most likely to benefit from immunotherapy is still something that has to be watched. For the treatment of patients who have cancer, immunotherapy checkpoint inhibitors are now being used and evaluated in either preclinical or clinical trials [33, 34]. This is because immunotherapy checkpoint inhibitors are an essential part of the immunotherapy strategy. The immunological milieu of the tumors is highly infiltrated with immune cells and contains a wide variety of immunomodulatory chemicals. This may have a significant bearing on the immunotherapeutic resistance and efficacy of the disease. We discovered that high-risk groups with shorter OS exhibited lower scores in aDCs, DCs, pDCs, and iDCs. These findings implied that an imbalanced and dynamic immune modulation was involved in the progression of LUAD. In addition, the elevated levels of T cell exhaustion markers that are brought about by continuous antigenic stimulation can result in the functional loss of CD8+ T cells [35, 36]. The expression of multiple immune checkpoints was significantly higher in the low-risk group, such as BTLA, CD28, and CD86. While the expression of PD-1 was higher in the high-risk group, it may indicate that the high-risk group had a better response to anti-PD-1 immunotherapy. Further, the TIDE score of high-risk group was lower than the low-risk group, also indicating the high-risk group had a better response to anti-PD-1 or anti-CTLA4 immunotherapy. Thus, a potential therapeutic approach for LUAD may consist of combining immunotherapy with the targeting of Cytochrome c-related ICD.

A key contributor to unfavorable clinical outcomes in cancer patients undergoing chemotherapy is the development of acquired resistance. If genes of sensitivity to anticancer treatments could be identified, it would be possible to improve the antitumor efficacy of chemotherapeutic drugs while simultaneously reducing their hazardous side effects. Then, we inquired as to whether particular regulators have the ability to forecast how a drug will react in a patient and the possible treatments that are available for LUAD. We found that many of 12 signature CCRGs were sensitive to EGFR-TKI, such as CYP24A1, FA2H, FGF2, KRT8, and MKI67 were sensitive to Afatinib, ADRB2, CYP24A1, FA2H, FGF2, and MKI67 were sensitive to Dacomitinib, and SLC2A1, ADRB2, FGF2, and KRT8 were sensitive to Dasatinib. Our findings suggested the genes in our prognostic model can be used as targets to predict drug sensitivity.

However, our study has several limitations. Firstly, the data originated from two different datasets, each of which had a small sample size. Moving forward, more thorough analyses will need to be conducted using large-scale and independent cohorts. Secondly, it should be noted that the clinical information that is offered by the TCGA data set is insufficient. There is a lack of knowledge regarding stages and grades for some individuals, in addition to a lack of data regarding treatments such as surgery, radiation, or chemotherapy. Thirdly, on the basis of investigations conducted in vitro and in vivo, the underlying molecular processes of LUAD that were investigated in this work have not been determined. Additional studies are necessary to broaden our understanding of cytochrome c and to foster the developments of novel therapeutic methods for LUAD patients.

## 6. Conclusion

In summary, we developed a reliable CCRGs genes signature that is capable of accurately assessing the clinical outcome of LUAD patients. Besides, we identified the immune microenvironments and immune targets were different between risk groups, which may be an explanation for unfavorable outcome in the high-risk group. In the meanwhile, our research might identify potentially useful targets that can boost the efficiency of cancer immunotherapy.

## Data Availability

The data are available from the corresponding author upon request.

## Supplementary Materials

Supplementary Materials Table S1 781 cytochrome c-related genes.
