# Supplementary material for: Application of Cytochrome C-Related Genes in Prognosis and Treatment Prediction of Lung Adenocarcinoma
Source: Dis Markers. 2022 Oct 3;2022:8809956. doi: 10.1155/2022/8809956 (PMC9550516; doi:10.1155/2022/8809956)
Supplement: Supplementary Materials — Table S1 781 cytochrome c-related genes. [file 8809956.f1.docx]

**Table 1** 781 cytochrome c-related genes.

Gene names

SCO2

MT-CO1

POR

COX15

COX10

CYCS

MT-CO3

COX4I1

COX6B1

SURF1

MT-CO2

COX5A

SCO1

PROC

COX8A

COA3

COX6A2

MT-CYB

CYP3A4

TACO1

CYBA

COA6

CYP2C9

TYMP

CYP2C19

COA8

CYP1A2

UQCRQ

CYP2D6

CYP1A1

CYB5R3

FANCC

UQCRFS1

NCAPH2

CYB5A

COA5

COX14

CYC1

UQCRB

CYP2E1

UQCRC2

PET100

COX6A1

NCF2

CYP3A5

COX7A2

CYP2B6

CRP

CYP2C8

F5

CYP2A6

CCR5

CYP1B1

COX20

PRKCD

CYBB

CYP17A1

LRPPRC

PRKCA

COX4I2

XPC

COX5B

UQCRC1

MT-TN

TP53

COX7A1

BCL2

MYC

PLEKHG5

BCS1L

IL6

COX7C

CYP19A1

UQCC3

COX7B

UQCC2

TNF

CST3

COX6C

AOPEP

UQCR10

CYP2C18

BAX

PLCG1

APOC2

SFTPC

NCF4

YARS1

FASTKD2

IFNG

COX17

CYP3A7

COX6B2

COX8C

CYP21A2

PRKCB

UQCRH

MT-TL1

CASP3

MAPK8

KIT

CYP2J2

RAF1

CASP9

UQCR11

PSAP

COX7B2

CYP11B2

PRKCZ

NDUFV1

CYP11A1

LMNA

FOS

JUN

PRKCE

NDUFA4

COX11

PET117

SDHB

NCF1

COX18

COX7A2L

PRKCG

HBB

INS

CXCR4

HCCS

APOE

COX19

APOC3

BRAF

NPC2

ABCB1

IL10

CYP4F2

COX7A2P2

CYP26A1

CYP4A11

SRC

CXCL8

PRKCI

COX16

CXCL10

MET

CASP8

UQCC1

CCL5

AKT1

CYP51A1

PLCB4

MAPK1

PRKCH

CYP11B1

PTPRC

CCL2

EGFR

BCL2L1

PLCE1

CYP7A1

CYP27C1

CYP2A13

CYP27A1

CYP24A1

CYP4F3

CYP26C1

CYP27B1

IGF1

COA1

VEGFC

PPIG

CYP4B1

ALB

CYB5R4

F2

CYP20A1

MIR122

CYP2A7

SERPINC1

CXCL12

APAF1

CYP3A43

ABCC8

TYR

CCR7

HLA-DRB1

LPL

PLCD3

NR3C1

CYP4F12

ABL1

CYP2F1

HLA-B

CYP2R1

ESR1

PLCH1

POLG

TWNK

CYP26B1

GSTM1

CXCR3

TGFB1

CYP7B1

CCR3

CYP4F11

CYP2S1

IFNL3

ABCC9

BID

CCL3

APP

LIPC

IFNA1

SLC25A4

EGF

CYP2W1

THBD

CYP4F8

CCR1

CYB561

BRCA1

ABCC2

MTHFR

CYP46A1

APOA1

CYP2U1

CSK

TRMU

IL1B

STAT3

CXCR2

ERBB2

ATP5F1A

FAS

CYB5R2

UQCRFS1P1

NQO1

COA4

INSR

NOS3

SMPD1

VEGFA

CD96

AHR

CYP4V2

TRPC6

MT-TE

COA7

RACK1

CYP8B1

PRKD1

CYBRD1

UQCRHL

LOC106029312

CYP4F22

IGF1R

SOD1

UBC

ABCC1

CYB5R1

HGF

CYB5B

TNFRSF1A

CEBPB

RET

PPARG

CXCL2

PTGS2

AIFM1

NCF4-AS1

CAT

CYP4Z1

MT-ATP6

CTNNB1

CFTR

CTSC

MPO

SDHC

COX8BP

CYP4A22

CYP4X1

BRCA2

GSTP1

NOS2

PARP1

HSPG2

PIK3CA

CEBPA

HFE

CCL11

HNF4A

MTOR

MDM2

GSTT1

BAK1

AR

NR1I2

CCR8

MMP9

MAPK14

COX6A1P2

SYK

PTEN

CYP39A1

COX6A1P1

MAPK3

GPT

MT-ND5

CXCL1

IL1RN

HRAS

CYP2D7

POLG2

UBE2C

RRM2B

ICAM1

DDIT3

TERT

IL1A

CDKN1A

OXA1L

CYCTP

CLEC7A

MYB

JAK2

TNFSF10

FASLG

IL4

QDPR

DIABLO

SOD2

COX6CP1

IL2

COX5AP1

COX7CP1

KRAS

MAPK10

COX7A2P1

KRT18

CCL8

HSPB1

POMGNT2

COX6B1P1

COX6B1P4

COX6B1P2

HIF1A

RASSF1

AGK

UGT1A1

FGF2

CYP2C23P

KCNJ11

NR3C2

PIK3CG

FGFR2

SLC2A1

CMC1

TBXAS1

RYR1

CCL13

RAC1

ABCA1

CTRC

KITLG

UCHL1

EPO

COX6B1P3

MIF

PPARA

CD4

COX5BP1

JAK1

CEL

SP1

CTLA4

POMC

PTPN11

ARAF

CCL27

G6PD

SLC17A5

XDH

CYB5RL

CD36

VKORC1

PCNA

COMT

LDLR

HMOX1

HNF1A

EIF2AK2

IFI27

GGT1

HSP90AA1

ATM

CCND1

MAP2K1

LEP

LYRM7

VDR

ANXA5

ABCC3

IL3

ETS1

MCL1

HLA-A

MMP2

CCL24

CAV1

BIRC5

NAT2

TLR4

CASP1

APOC1

CYB561D2

COX5BP6

CXCL6

SERPINE1

FYN

CDKN3

CXCL14

STAT1

PTK2

EPHX2

BMPR1B

ABCC4

APOB

NPM1

CYB561A3

COX4I1P1

IL18

SHC1

VWF

CMC2

BAD

CLEC11A

KRT8

MBL2

CENPC

EP300

COX5BP2

COX7BP2

COX5BP7

COX4I1P2

COX5BP3

COX5BP4

COX5BP5

CYP2C64P

DGKQ

CYP2C61P

CYP2C58P

CYP2C59P

CYP2C60P

CYP2C56P

CYP2C63P

CYP2C115P

NLRP1

UQCRHP1

MAPK9

NFIC

FLT4

SCARB1

PSEN1

RAD51

ABCC6

CHUK

ETHE1

NFKB1

APOA5

GBA

ACE

TTC19

CCNC

XIAP

ESR2

MMP1

CSF3

MEFV

FGFR1

NOS1

CETP

PDGFRB

FANCA

ADIPOQ

EDN1

IFNB1

PPIC

E2F1

FANCD2

UQCRC2P1

ERCC1

COX7BP1

COX5AP2

COX6B1P6

COX6CP2

COX6CP7

COX6A1P3

COX6B1P5

COX6B1P7

COX6CP11

COX6CP12

COX6CP13

COX6CP15

COX6CP16

COX6CP17

COX6CP18

COX6CP4

COX6CP5

COX6CP6

COX5BP8

COX6CP10

COX6CP14

COX6CP3

COX6CP8

LOC107985222

COX6CP9

ERCC6

IL6R

CDK4

KDR

CDK1

CFLAR

ABCC5

F10

TF

CASP2

VCAM1

TK2

PUS1

RB1

CTSD

NR1I3

PTK2B

PIK3R1

PNLIP

IGF2

TGFA

NTRK1

CREB1

MYBPC1

BIRC3

PLA2G2A

IRF3

AFP

CSF2

CREBBP

ERCC2

CYP21A1P

PRKCSH

RHOC

CASR

ATP5MC1

MTCO2P12

CYB561D1

NDUFB8

OPA1

VAV2

AKAP13

CD40LG

GAPDH

EPHX1

CYP4Z2P

UQCRHP4

UQCRBP3

NR2C2

PRTN3

LDHC

CASP7

MUC1

HSPA4

UQCRFS1P2

ARHGEF2

SLCO1B1

CCNB1

CYP3A51P

UQCRBP1

UQCRBP2

UQCRHP2

ENSG00000232135

UQCRHP3

UQCRFS1P3

LOC727947

ENSG00000228330

ENSG00000231034

ENSG00000250214

ENSG00000233114

ENSG00000256148

ENSG00000256984

ENSG00000231906

ENSG00000235449

ENSG00000233444

ENSG00000251105

ENSG00000230609

ENSG00000270868

ENSG00000271749

ENSG00000254478

LOC100421855

ENSG00000218483

ENSG00000219604

ENSG00000220139

ENSG00000220326

ENSG00000223544

ENSG00000250984

LOC692246

ENSG00000234041

ENSG00000234863

ENSG00000239568

ENSG00000225583

ENSG00000225911

ENSG00000226324

ENSG00000227979

ENSG00000230112

ENSG00000230119

ENSG00000249829

ENSG00000242152

ENSG00000248770

ENSG00000271440

ENSG00000282852

ENSG00000270718

ENSG00000270902

ENSG00000254373

ENSG00000254437

ENSG00000256098

ENSG00000257296

ENSG00000260764

ENSG00000270620

LOC100288010

LOC100288385

LOC100289479

LOC100506672

LOC100507586

ENSG00000258205

LOC107987328

LOC107987329

LOC112268032

ENSG00000225183

ENSG00000235463

ENSG00000249780

LOC107986878

ENSG00000223986

ENSG00000231961

ENSG00000234594

ENSG00000242036

ENSG00000225551

ENSG00000227004

ENSG00000234108

ENSG00000240103

ENSG00000250213

ENSG00000250839

ENSG00000226014

ENSG00000229899

ENSG00000233648

ENSG00000248652

LOC107985982

LOC107984162

ENSG00000268747

ENSG00000275515

ENSG00000284327

ENSG00000288680

ENSG00000271600

ENSG00000285142

ENSG00000254914

ENSG00000257070

ENSG00000260639

ENSG00000270381

LOC107986374

LOC107986039

LOC107985802

ENSG00000224518

ENSG00000250890

LOC107985114

LOC107985645

LOC107985265

LOC107985453

LOC107984365

ENSG00000284591

ENSG00000275130

ENSG00000283339

ENSG00000283657

ENSG00000253261

ENSG00000283356

ENSG00000283576

ENSG00000257570

DRD2

F3

CYB5D2

LIPG

NFKBIA

CLEC3B

CDH1

RPS27A

MX1

PON1

C1QC

MB

PLA2G4A

IRS1

MT-TS1

CDC20

MAP2K4

PRNP

TGFBR1

DGAT1

SFTPB

HMGCR

BCL2L11

SST

GCK

BECN1

ROCK1

BMP1

PLA2G6

POLR1C

HBG2

HLA-DQB1

CD40

NFE2L2

TTR

PROS1

LIPE

FDX1

APC

PGR

DDX3X

ABCG2

NLRP3

NUDC

SPP1

FANCF

PDGFB

CYB5D1

CTSB

CSF1

IL2RA

PDGFRA

NR1H2

YARS2

LYN

SNCA

BMP6

LCK

PRKN

IL1R1

PTGIS

LDHD

TLR2

TXN

CALCA

EGR1

ALDH2

OLR1

GLC3C

ATP5MC3

GH1

ITGB1

BDNF

AGTR1

UCP3

UCHL3

LCAT

MKI67

CACNA1C

CMC4

FA2H

CDK2

SKP2

PLAU

AGT

TGM2

CLU

ADRB2

CYP2T1P

VAV3

TRPC1

PTPA

CDKN1B

BGLAP

HTR2A

GZMB

CD44

MT-ND1

CYBC1
